# Ophthalmologic Comorbidities in Alopecia Areata

**DOI:** 10.3390/jcm14238409

**Published:** 2025-11-27

**Authors:** Piedad M. Guavita Falla, Diego Buendía-Castaño, Ángela Hermosa-Gelbard, Bárbara Burgos-Blasco, Patricia Burgos-Blasco, Sergio Vañó-Galván, David Saceda-Corralo

**Affiliations:** 1 Dermatology Service, Pedro de Valdivia RedSalud Medical Center, Av. Nueva Providencia 1920, Metropolitan Area, Providencia 7500000, Chile; 2 Dermatology Department, Instituto Ramón y Cajal de Investigación Sanitaria—IRYCIS, Ramon y Cajal Hospital, 28034 Madrid, Spain; 3 Hair Disorders Unit, Grupo Pedro Jaen, 28002 Madrid, Spain; 4 Ophthalmology Department, Hospital Clínico San Carlos (IdISSC), 28040 Madrid, Spain

**Keywords:** alopecia areata, comorbidity, ophthalmology, immune privilege

## Abstract

Alopecia areata (AA) is a complex disease with a multifactorial etiology, in which autoimmune mechanisms play a central role. Increasing evidence suggests that AA may be a systemic condition, potentially affecting organs beyond the skin due to shared pathogenic pathways. One proposed mechanism is the breakdown of immune privilege, a protective state that limits immune activity in specific tissues, such as the hair follicle and the eye. Although research on the relationship between AA and ophthalmic comorbidities remains limited, several studies have reported recurrent ocular abnormalities, whether subclinical or symptomatic, appearing at younger ages than typically observed in the general population. This review aims to summarize current knowledge on the association between AA and ocular involvement, exploring shared pathogenic mechanisms, clinical eye manifestations, and practical considerations for addressing ocular symptoms in dermatological practice.

## 1. Introduction

Alopecia areata (AA) is an organ-specific autoimmune disease characterized by a variable course, usually of recurrences alternating with remissions, and a wide spectrum of manifestations [1]. After androgenetic alopecia, it is the second most frequent cause of non-scarring hair loss. It affects 2% of the general population at some point in life, regardless of gender, ethnicity or age [2,3]. Additionally, this disease is known to have a considerable impact on health-related quality of life [4,5].

AA is associated with several autoimmune diseases such as thyroid disorders, inflammatory bowel disease, systemic lupus erythematosus, rheumatoid arthritis, psoriasis, and vitiligo [6,7,8]. Other comorbidities described in the literature are atopy (allergic rhinitis, asthma, and atopic dermatitis), iron deficiency anemia, vitamin D deficiency, mental disorders (depression, anxiety, alexithymia, and obsessive–compulsive disorder), diabetes mellitus, dyslipidemia, and hypertension [8,9,10]. Less explored have been ocular and audiological abnormalities, with limited studies demonstrating their presence in the context of AA [10,11,12].

This review synthesizes the existing evidence on ophthalmologic involvement in AA, from its possible etiopathogenic connection, through the ocular clinical manifestations described in patients with this disease in different studies from the 1960s to the present, and concluding with a series of recommendations on the initial approach for dermatologists in clinical practice regarding screening for eye conditions in patients with AA.

## 2. Materials and Methods

We conducted a narrative review of the literature in the PubMed, MEDLINE, ScienceDirect, and Google Scholar databases, to summarize the literature on the etiopathogenesis of AA and eye diseases, as well as the presence of the latter in AA. We used various combinations of MeSH terms such as “alopecia areata”, “ophthalmology”, “eye”, “eye diseases”, “ocular”, “immune privilege”, as well as the different ocular areas (e.g., eyelids, eyebrows, eyelashes, conjunctiva, cornea, lens, retina, among others). The inclusion criteria were as follows: English and Spanish languages, empirical research and review articles to contextualize and support our findings on immune privilege and other causal factors of AA and ocular diseases, and finally, studies with different methodological designs (e.g., case reports, case series, cross-sectional studies, case–control studies, cohort studies) to document our findings about ophthalmologic comorbidities in AA. The search yielded a total of 82 articles on the etiopathogenesis of AA and eye diseases, and 140 articles on the presence of ocular comorbidities in AA, all published between August 1963 and February 2025, with no duplicate articles. Zotero 7.0.27 was used as the reference management database. 

## 3. Etiopathogenesis of Alopecia Areata and Its Relation to Ocular Involvement

Although the exact cause of alopecia areata is not well understood, immunological factors are considered to play a pivotal role in genetically predisposed individuals, in whom certain environmental factors contribute to triggering the disease [1,2,3,13].

### 3.1. Immunological Factors: What Is Immune Privilege?

Since some organs have a limited capacity to regenerate, even in the setting of minimal inflammation, the human body developed a state of anergy, which means that the detection of antigens is restricted, thus protecting them from damage caused by immune recognition. This adaptive state is known as immune privilege (IP) [14].

Organs that have this IP are the eye, the placenta, the liver, the testes, the central nervous system and, thanks to several studies, it has been suggested that the hair follicle is also protected, in particular the bulb during the anagen phase and the bulge throughout the follicular cycle [14,15,16,17].

#### 3.1.1. IP in the Hair Follicle

Several mechanisms involved in the generation and preservation of hair follicle IP have been described, which can be grouped into physical barriers, “sequestration” of autoantigens and reduction in immune activity [13,14,18].

As for physical barriers, the epithelial hair bulb lacks lymphatic drainage and is enveloped by a special barrier of extracellular matrix with expression of specific glycosaminoglycans, all of which prevents the trafficking of immune cells to this area [15,18].

The absent or low expression of classical major histocompatibility complex class I (MHC-I) molecules serves to “sequester” or hide autoantigens associated with melanogenesis and/or the anagen phase and thus prevents their presentation to CD8+ T lymphocytes (CD8+ TL) [18,19]. Likewise, expression of non-classical MHC-I molecules, such as HLA-E and HLA-G, has been documented to be associated with suppression of CD8+ TL- and natural killer (NK) cell-mediated lysis [18].

In relation to the reduction in immune activity, there is local production of potent immunosuppressants such as interleukin-10 (IL-10), transforming growth factor β1 (TGF-β1), and alpha-melanocyte stimulating hormone (α-MSH), among others [14,18]. These are the so-called IP guardians. Other mechanisms are the production of Fas ligand (FasL) that intervenes to eliminate Fas-expressing autoreactive T cells, and low or absent major histocompatibility complex class II (MHC-II) molecules on Langerhans cells [14,18,19]. In addition, the normal hair follicle suppresses NK activity by these processes: low expression of the activating receptor NKG2D (Natural Killer Group 2D Receptor) along with increased inhibitory receptor KIR (Killer-cell Immunoglobulin-like Receptor) on NK cells, production of macrophage migration inhibitory factor (MIF), and decreased or absent expression by the hair follicle of NK cell ligands such as MICA (MHC class I chain-related protein A) and ULBP3 (UL16-binding protein 3) [14,18,19,20].

#### 3.1.2. Ocular IP

The mechanisms of IP in the eye are similar to those described above for the hair follicle. As for the physical barriers, the cornea is avascular, lacks lymphatic vessels, presents a constant regeneration of the epithelium, and a protective layer formed by tight junctions. Similarly, there is a blood–retinal barrier that also has tight junctions formed by retinal pigment epithelial cells [21,22].

Sequestration or concealment of autoantigens has also been demonstrated, which is achieved by the aforementioned physical barriers, the absence of MHC-II on antigen-presenting cells, and the low expression of classical MHC-I molecules in ocular tissues [21,22,23]. Furthermore, the presence of immature myeloid leukocytes in the cornea facilitates the induction of immune tolerance on the ocular surface [24].

In terms of reducing immune activity, aqueous humor and vitreous fluid have anti-inflammatory properties. For example, aqueous humor components suppress antigen-induced lymphocyte proliferation in vitro and inhibit the expression of local delayed-type hypersensitivity (DTH) responses [24]. On the other hand, ocular parenchymal cells express FasL which triggers apoptosis of inflammatory cells and, moreover, there is specialized regulatory T-cell (Treg) activity that suppresses Th2 and Th17 lymphocyte-mediated inflammation [24,25].

Other factors described are inhibition of NK cells and presence of local immunosuppressants such as TGF-β2, α-MSH, vasoactive intestinal peptide (VIP), MIF and calcitonin gene-related peptide (CGRP) in the aqueous humor [23,24,25].

Two additional mechanisms have been identified: the presence of complement regulatory proteins in the aqueous humor, vitreous fluid and corneal epithelium, and the ACAID [24,25]. ACAID stands for anterior chamber-associated immune deviation, which is the best studied model of ocular immune tolerance and is regarded as the systemic element of IP, as it involves spleen activity. This phenomenon is characterized by downregulation and suppression of Th1 pathways in response to a foreign protein in the anterior chamber of the eye, and manifests itself in different ways, such as through attenuated systemic DTH responses, stimulation of non-complement-binding IgG1 antibodies, and production of antigen-specific immunomodulatory cells in the spleen (Tregs, marginal zone B cells, γδ Tregs, iNKT, and NK regulatory cells). These cells spread throughout the body and induce antigen-specific immune deviation [21,23,24,25,26].

It should be noted that IP in hair follicle and eye involves not only specialized local mechanisms that protect against inflammation in situ but also, at least in the case of the eye, includes mechanisms that regulate global immune responses, as we have just seen with the ACAID phenomenon [18,25,27,28]. Systemic induction of a tolerogenic response to hair follicle/ocular antigens by induction of Tregs, apoptosis of antigen-specific thymic and peripheral T cell clones, and T cell receptor desensitization are further examples of this [18,27,29,30]. All of the aforementioned may also imply that the singular environment at these immunoprivileged sites may impact overall immune responses if tolerance is lost.

A comparative summary of IP in both hair follicle and eye is shown in Table 1.

#### 3.1.3. Loss of IP in AA and Its Connection to Ocular Comorbidities

The main pathophysiological hypothesis of AA is based on the failure of the IP in the anagen hair bulb [14,18,19,20,23]. However, although this is considered a key condition, it is not sufficient for the development of the disease, and other requirements (autoimmune factors and/or non-immune triggers) must be met for AA to occur [18,31,32]. These circumstances may occur simultaneously or in isolation, as shown in Table 2 [18,31,32,33].

Induced by all or any of the situations referred to in Table 2, the IP collapses, which means that it loses its ability to induce tolerance and immunoinhibition. This collapse of the IP is required as a final condition for AA to develop, which is characterized by the fact that only hair follicles in anagen III-VI are attacked (period in which mature autoantigens are generated) and, as a consequence, premature termination of the anagen phase ensues, with consequent follicle dystrophy [18,31,32,33]. Interestingly, besides causing IP collapse, interferon-gamma (IFN-γ) is a potent inducer of catagen in normal human anagen hair follicles [34].

It is important to emphasize that IP loss is a complex phenomenon and is therefore not only due to autoimmune factors, but, as described in Table 2, non-immune triggers may also play an important role (e.g., excessive secretion of hair follicle IP collapse inducers in an antigen-non-specific way) [18,31,33,35]. Moreover, as previously mentioned, IP is not only a local phenomenon, but also a systemic one, and its collapse could potentially impact the global immune response, affecting both the immunoprivileged organ and probably other tissues [18,25,27,30,36].

##### What Is the Phenomenon That Connects AA to Ocular Involvement?

Both the hair follicle and the eye have IP, and the way in which this protection is carried out has similarities (Table 1). Under this premise, it is postulated that IP collapse, usually generated by one or all of the aforementioned pathogenic conditions, is a fundamental factor for the development of AA and ophthalmologic pathologies [23,37]. Therefore, although more studies are needed in this field, it is considered that in AA the occurrence of ocular comorbidities may be due to pathophysiological phenomena common to both entities, such as TL-mediated autoimmune activity [38,39,40,41], or the development of inflammatory events, still considered emerging, of a non-autoimmune nature (e.g., oxidative damage, role of substance P, neurogenic inflammation, dysbiosis) [18,41,42,43]. There are several clinical examples of ocular involvement in AA with some autoimmune intervention, such as decreased lacrimal secretion [39,42], or impairment of the retinal pigment epithelium (RPE), among others [12,41,44].

Whether the same insult would act to affect different cells in the hair follicle and the eye or whether inflammation in one of these tissues would trigger disease in the other organ remains to be determined [39,44,45]. Likewise, it must be pointed out that the causal correlation between ocular and follicular inflammation has not yet been fully established and therefore remains an area of great interest for further research.

It is crucial to remember that ocular IP is found in several areas such as the cornea, anterior chamber, iris, ciliary body, vitreous humor and retina [21,22,23,24,25,26,37,46,47]. Therefore, IP collapse is a potential factor in the genesis of many of the ocular diseases in AA as will be shown below.

### 3.2. Other Factors in the Pathogenesis of AA and Ocular Comorbidities

Susceptibility to develop AA is considered to be hereditary, but its occurrence is probably triggered by environmental factors and the same is postulated in the genesis of associated eye diseases [13,42]. Genome-wide association studies have found that many of the genes involved in AA are also present in other autoimmune conditions, including ocular ones [13,42]. Consequently, susceptibility loci involving human leukocyte antigen (HLA) regions have been identified, as well as genes associated with functions of innate and adaptive immune responses, such as regulation of self-antigen expression, negative selection of autoreactive TL at the thymic level, Treg differentiation/activity, apoptosis, proinflammatory cytokine production, and chemotaxis, among others [13,48,49].

Regarding systemic inflammation (SI) and its influence on ocular involvement, it is relevant to recognize that IP collapse is not the only feature that could contribute (remember that local and systemic elements intervene in IP). Since SI is multifactorial, it is crucial to highlight that genetic influence, infections, and concomitant autoimmune/non-autoimmune diseases may be implicated in eye abnormalities found in AA [13,28,29,30,50,51,52]. In addition, according to several studies, AA is associated with systemic inflammation, as evidenced by increased cardiovascular, atherosclerotic, and peripheral immune biomarkers, and a possible correlation with an increased risk of heart disease and stroke [53,54,55,56,57].

Among other conditions that can potentially generate ocular compromise in AA is treatment itself. For instance, corticosteroids, commonly used in AA, are able to generate ocular alterations in any of their routes of administration (topical, oral, etc.). The main complications at this level are cataracts and glaucoma [3,12,58]. As for cataracts, they are more likely to be of the posterior subcapsular type, occur with doses higher than 10 mg/day of prednisone or its equivalent for over a year, and children are more susceptible [12,58,59]. Glaucoma is more likely with topical periocular steroid use and with a minimum of 2 weeks of treatment [58,59,60]. On the other hand, topical application of prostaglandin analogs, an alternative in the treatment of eyelash AA [3,61,62], can cause conjunctival hyperemia, dry eye (due to tear film instability), darkening of the iris, pigmentation of the periocular skin, and seldom cystoid macular edema, anterior uveitis, reactivation of herpes simplex keratitis, or iris cysts [63,64,65].

Another factor implicated in the onset and progression of AA and ophthalmologic comorbidities is oxidative stress. Patients with AA have higher levels of lipid peroxidation products and nitric oxide (NO), lower activity of the antioxidant enzyme superoxide dismutase, and it has been suggested that oxidative stress interferes with follicular cycle [66,67]. Along the same lines, the lens is under constant photooxidative stress due to its light filtering function, which, coupled with a decrease in the activity of the enzymes glutathione peroxidase and glutathione S-transferase (GST) when exposed to ultraviolet radiation, may increase the likelihood of cataract formation [68,69]. Given that cataracts have been described in patients with AA with a higher frequency than in the general population, and in some studies independently of other risk factors (e.g., atopy, systemic steroid therapy) [40], it would be interesting to clarify the role of oxidative stress in this clinical background.

Furthermore, it should be noted that skin, hair and lens are derived from the ectoderm, so this common embryological origin could also contribute to the development of cataracts in AA [12,41]. This shared origin also explains why certain conditions, such as ectodermal dysplasias, can manifest with hypotrichosis, eye abnormalities (e.g., dry eyes, cataracts, corneal dysplasia), and other alterations of ectodermal tissues [61,70].

On the other hand, the role of environmental conditions such as psychological stress seems to play a role in some patients with AA, although this association remains controversial due to disparate results of clinical studies, with some finding no significant relation with the onset of the disease [71,72,73], and others suggesting it as a possible triggering or aggravating circumstance, given that certain patients report stressful events prior to the onset of AA symptoms [74,75]. In support of the latter theory is the impact of neuroendocrine factors such as substance P (SP), a neuropeptide mentioned above (Table 2), which is released from perifollicular sensory nerve fibers, is recognized for inducing hair follicle IP collapse, and whose production has been linked to psychoemotional stress [18,31,76,77,78]. Similarly, the participation of substance P in the homeostasis of the corneal epithelium, whose alteration would cause corneal lesions and dry eye disease, as well as in the pathogenesis of uveitis, has been demonstrated in several clinical and experimental trials [24,76,79,80].

Another point to consider is the association of AA with other diseases, which in turn will influence the development of ocular pathology. For example, let us look at the relationship between atopic dermatitis and AA [8,9,10,81], and also consider that atopic dermatitis has traditionally been associated with keratoconus, blepharitis, and conjunctivitis [82,83,84,85]. As we will see below, there is further evidence for the occurrence of these specific eye diseases in AA. Whether atopic dermatitis is the main risk factor for the development of keratoconus, blepharitis, and conjunctivitis in patients with AA or whether AA is an independent condition for these ocular pathologies is a question to be clarified in future research.

**Figure 1:**
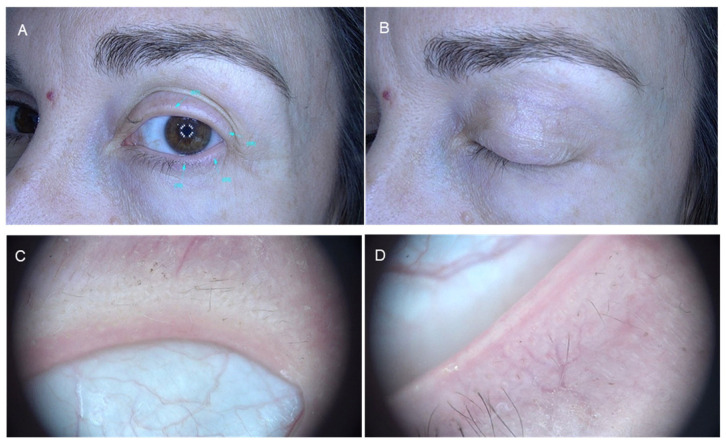
Eyelash madarosis in alopecia areata. Panels (**A**,**B**): Clinical presentation. Panels (**C**,**D**): Dermatoscopic images.

Finally, madarosis in AA can act as a mechanical feature that would add to the etiology of ocular diseases (Figure 1). Madarosis is defined as the total or partial loss of eyebrow or eyelash hair. Milphosis is another term that refers specifically to eyelash loss [61,86]. Eyebrows and eyelashes provide protection for the ocular area from sweat, microorganisms, dust, irritants, and even light, wind, and water [87,88]. In alopecia areata, the absence of eyebrows and/or eyelashes is a source of ocular irritation leading to frequent eye rubbing, and the entry of exogenous substances into the ocular surface which, in turn, will participate in the development of blepharitis, dryness, keratoconus and keratitis, including others [61,87,89].

## 4. Clinical Manifestations of Ocular Involvement in AA

Ophthalmologic manifestations in patients diagnosed with AA have been described in numerous studies with various methodological designs, such as case reports, case series, cross-sectional studies, case–control studies or cohorts, with varied findings in both the anterior and posterior segments of the eye.

Table 3 presents the most relevant publications from the 1960s to the present day, ordered by ocular anatomical area, from anterior to posterior segments, accompanied by a section of comments in the third column (see Supplementary Material for this section) on hypotheses about pathogenesis of AA and ophthalmic comorbidities, clinical recommendations (both according to authors of each article) or specific observations regarding the respective study (the latter from our perspective). Some papers are repeated by anatomical area due to findings of more than one clinical manifestation in them. This is only a descriptive table and, therefore, it does not intend to establish etiological associations between AA and ocular findings, so it strictly adheres to what is mentioned in each study by its authors regarding results, causal hypotheses, and patient follow-up.

**Figure 2:**
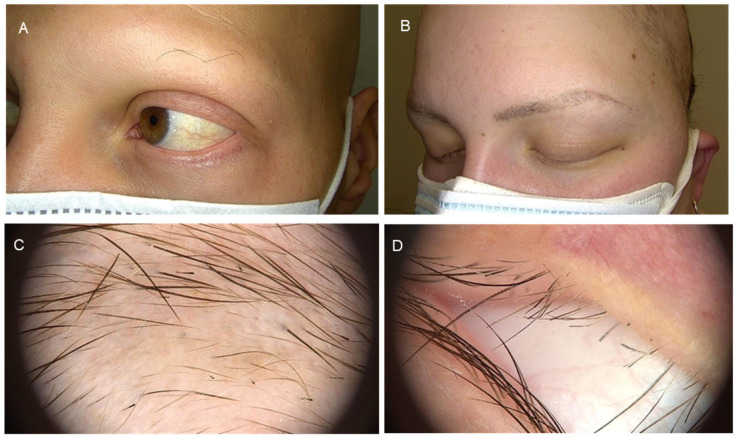
Eyebrow and eyelash alopecia areata. Panels (**A**,**B**): Clinical presentation. Panels (**C**,**D**): Dermatoscopic images.

The first part of Table 3 refers to the involvement of eyebrows and eyelashes, which is considered relevant as part of any dermatological and ocular examination. A detailed evaluation of the periocular skin as well as the eyelids is vital in the initial approach, and it is important to be aware that the presence of madarosis can be part of many nosologic entities. Eyebrow and eyelash AA may be underestimated in many cases and has not been widely reported in the literature as a separate entity (Figure 2).

From Table 3, we can see that there are multiple and diverse ophthalmological manifestations described in the literature, based on studies with different methodological designs.

At the periocular level, eyebrow and/or eyelash madarosis is the most frequently observed condition [90,91,92,93,94,95,96,97,98,99,100,101,102,103,104,105,106]. Subsequently, if we consider the anterior segment of the eye, we can observe that blepharitis, conjunctivitis, dry eye disease (DED), iridocyclitis, and lens abnormalities are the most common conditions in patients with AA [12,39,40,42,100,102,105,108,109,110,111,112,113,114,115,116,117,118]. With regard to conjunctivitis, in most studies it presented features (conjunctival papillary hypertrophy) or was clearly of the allergic type [39,42,110,113]. Furthermore, dry eye symptoms and/or direct evidence of DED based on clinical parameters (ophthalmological examination and miscellaneous tests), was quite common in the AA context, and it is suggested that this condition may be due to alterations in tear stability due to the loss of goblet cells, which in turn arises from the interplay of multiple elements, such as chronic inflammation, autoimmunity, genetic predisposition, and environmental influences [39,42,102,105,108,112].

On the other hand, the most consistently reported lens abnormalities were asymptomatic opacities of various characteristics [12,40,41,105,109,116,117,118], with punctate opacities being the most common, and cataracts. Regarding this last finding, posterior subcapsular and cortical cataracts were the most frequently encountered [40,41,105,115].

It should be noted that, in addition to IP loss, there may be other risk factors involved in the etiology of cataracts in AA, such as oxidative stress, a common embryological origin with the hair follicle (ectoderm), steroid treatment, and atopy [12,40,41,58,66,67,68,69].

Although it has been described in few studies, it is worth mentioning that there may be an increased risk of keratoconus in patients with AA [110,112], which may be influenced by factors such as eye rubbing, keratoconjunctivitis, and autoimmune diseases.

Furthermore, when addressing findings in the posterior segment of the eye, it is evident that changes in choroidal thickness, abnormalities of the retinal pigment epithelium (RPE), and degenerative and vascular changes in the retina, were the most consistently reported [12,39,40,41,100,105,108,109,117,119,120,121,122,123].

In terms of choroidal thickness, it has been observed that in newly diagnosed cases of AA, the choroid is significantly thicker [120]. However, it is suspected that this thickness could change over time, with the progression of the disease, its severity, and/or the presence of poor prognostic factors (nail involvement, early onset AA, diffuse involvement, positive family history for AA, positive thyroid autoantibodies) [119,120].

RPE abnormalities are variable and range from hyperplasia to reticular degeneration, pigmentary clumping, and macular changes in the RPE, including others [12,39,40,41,44,100,105,108,109,117,121,123]. Degenerative changes in the retina described in several studies are also prevalent and vary in their presentation: drusen, macular degeneration, and lattice degeneration, to name a few [12,39,40,41,100,105,108,117,121,123]. Lastly, retinal vascular changes differ in their manifestations, with hyalinized vessels, acute hemorrhagic retinal vasculitis, and retinal vascular occlusion being reported [100,122,123].

To conclude this overview, we have refractive errors, with myopia and hypermetropia being the most frequently reported [41,105,109,128,129].

All things considered, it is still pertinent to note that study results are heterogeneous regarding ocular manifestations in AA, and some of them find no significant differences compared to controls in selected clinical features [40,108,118,119,120]. In the same way, the current evidence has limitations that should be kept in mind when interpreting its results, such as small size of some samples, retrospective designs, and potential confounding factors in certain studies (e.g., atopy, corticosteroid use, associated autoimmune diseases).

**Figure 3:**
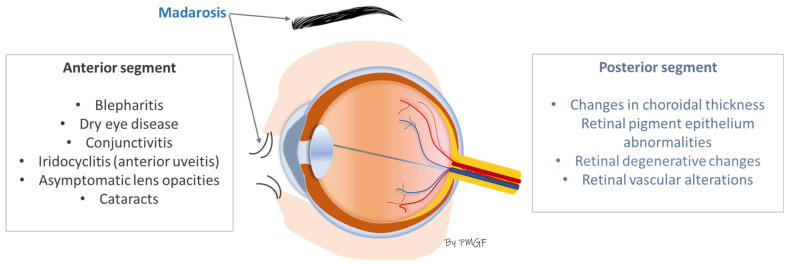
Most frequent ocular manifestations in patients with AA.

As the final part of this clinical section, Figure 3 summarizes the most common ophthalmological manifestations in patients with AA, as explained above.

## 5. What Would Be the Initial Approach to Ocular Comorbidities in Patients with AA in Dermatological Practice?

Recognition of ophthalmological conditions in patients with AA will only be possible if it is taken into account that such involvement is likely in this context. In follow-up visits for alopecia, a series of “key questions” can be incorporated to detect ocular symptoms, based on which it may be possible to identify the presence of eye conditions that should be evaluated or treated by other specialists.

### 5.1. General Key Questions Regarding Ocular Symptoms for Dermatologists

It is advisable to start with a broad approach, for which the use of general questions will allow for the more efficient detection of ophthalmological symptoms [130]:

Do you currently have any problems with your eyes?/Is there anything about your eyes that worries or concerns you?

Is the problem affecting one or both eyes?

Did it start suddenly or gradually?

Have your symptoms improved, worsened, or remained the same since the beginning?

Has your problem been intermittent or seasonal, or does it worsen at any time of day? If so, what makes it better or worse?

Have you had any eye problems in the past?

Have you had any eye surgery?

Are you taking any medications? Do you use any products/medicines for your eyes?

Has anyone in your family been diagnosed with eye diseases?

### 5.2. Specific Key Questions According to the Eye Condition Reported in AA for Dermatologists

Based on what we have detected in the general questionnaire, we propose to move forward with specific questions, starting with the structures of the anterior segment of the eye and continuing with those that make up the posterior segment.

Consequently, we have relied on the most common ophthalmological manifestations in patients with AA, as reported in the scientific literature cited in Table 3 and summarized in Figure 3, to organize these specific key questions that should be asked during the dermatological consultation, as shown in Table 4 and Table 5 [131,132,133,134,135,136,137,138,139].

### 5.3. Another Assessment Worth Considering Regarding the Evaluation of Ocular Involvement in AA

A valuable contribution to the comprehensive approach to eye disorders in AA would be to assess disease burden caused specifically by such involvement. In this context, patient-reported outcomes (PROs) are tools that could contribute not only to the detection of ophthalmic diseases (for example, through the reporting of symptoms) but also to timely treatment and improved quality of life. PROs are defined as outcomes reported by the patient where there is no clinical interpretation [140]. Simple descriptions of symptoms or composite outcomes, such as patient satisfaction or health-related quality of life, are ways to assess PROs [141,142]. Dermatology Life Quality Index (DLQI), Skindex-29, and Skindex-16 are some examples of validated PROs in dermatology [142,143]. Some of the specific PROs for alopecia areata that have been validated are the Alopecia Areata Patient Priority Outcomes (AAPPO) and the Alopecia Areata Symptom Impact Scale (AASIS) [144,145,146].

Several studies in AA have incorporated PROs into the initial examination and follow-up of these patients, and some of them have evaluated ocular findings using these measures [4,5,101,102,147,148,149]. Therefore, it would be a fundamental contribution if, in addition to the general and specific anamnesis proposed above, some PROs focused on eye symptoms in AA patients were included, mainly in new research and, hopefully, in the future and, as far as possible, in daily clinical practice.

### 5.4. Recommendations on Initial Ophthalmological Management, Multidisciplinary Approach, and Follow-Up

In this section, we include guidance on conditions affecting the ocular surface (blepharitis, dry eye disease and keratitis), which cause symptoms such as dryness, irritation, redness, and discomfort. Other common eye conditions reported in AA (e.g., cataracts, retinal disorders) should be finally confirmed and treated directly by an ophthalmologist, as their management exceeds the scope of this article.

In cases where there are mild ocular surface symptoms and no red flags, dermatologists may initiate treatment as part of the overall management plan. However, for more complex or severe cases, coordination with ophthalmology is essential to ensure comprehensive care.

For first-line management of ocular surface disease, eyelid hygiene is essential. Regular cleaning of the eyelid margin is recommended to remove debris, crusts, and bacteria. Warm compresses and gentle eyelid scrubs with products such as baby shampoo or commercial eyelid cleansers are effective options [137,150,151]. As for lubricants, preservative-free artificial tears should be used regularly to alleviate dryness and prevent ocular surface damage. In cases of persistent dryness, gel lubricants or ointments applied at night can provide longer-lasting relief [150,151]. Regarding environmental measures, patients should avoid dry, windy, or air-conditioned environments, and the use of humidifiers in dry settings may be beneficial [137,150]. In cases where conservative measures are insufficient, especially in patients with moderate to severe dry eye or those who do not respond to topical lubricants, topical treatments including cyclosporine, insulin or, autologous serum can be considered, although these must be prescribed by an ophthalmologist [150,151,152].

It is important to coordinate early involvement of ophthalmology when initiating systemic therapies for AA, such as corticosteroids, JAK inhibitors, or immunosuppressants, as these treatments may exacerbate or predispose patients to eye disease [58,59,153]. In this way, the onset of potential complications, such as elevated intraocular pressure, cataracts, or ocular surface diseases, can be monitored.

Concerning follow-up schedules, a comprehensive ocular surface examination should be performed at the start of systemic therapy to establish a baseline for the patient’s eye health. A follow-up visit should be scheduled 1 to 2 months after initiating systemic therapy to assess any changes. Once systemic therapy has stabilized, follow-up every 6–12 months may be appropriate, although any significant changes in eye symptoms or signs should prompt earlier reevaluation. Continued coordination between dermatology, ophthalmology, and other specialists is essential for comprehensive care, especially for patients undergoing long-term systemic therapy.

## 6. Conclusions

Patients with AA appear to be at higher risk of developing ocular involvement in different anatomical areas, as has been demonstrated in various studies. These manifestations range from madarosis, blepharitis, dry eye disease (DED), and conjunctivitis, to cataracts, choroidal thickness alterations, retinal pigment epithelium (RPE) abnormalities, and retinal degeneration or vascular changes. Some of these conditions, whether acute or chronic, may, in rare cases, lead to permanent visual impairment.

The relationship between ophthalmological disorders and AA has not been fully elucidated; however, the evidence to date supports a shared immunological basis (collapse of immune privilege) in genetically susceptible patients exposed to environmental triggers. In view of the above and as a precautionary measure, we recommend that dermatologists include a history of ocular symptoms, both through anamnesis and other measurements (e.g., PROs), preferably in all patients with AA. This would enable early detection of comorbidities and prevention of disease progression, ensuring referral to ophthalmology when appropriate.

## Figures and Tables

**Table 1:** Comparative characteristics of immune privilege at the hair follicle and ocular levels.

	Hair Follicle	Eye
***Distinctive features***	IP is a periodic phenomenon at bulb but remains constant at bulge level	Presence of ACAID: it involves DTH suppression, among other mechanisms
**Both organs possess:**	Physical barriers	
**(*common features*)**	Low expression of classical MHC-I molecules	
	Reduced expression of MHC-II in antigen presenting cells	
	Strong production of similar immunosuppressive molecules (e.g., IL-10, TGF-β1, α-MSH)	
	Induction of FasL-mediated apoptosis of immunocytes	

IP, immune privilege; ACAID, anterior chamber-associated immune deviation; DTH, delayed-type hypersensitivity; MHC-I, major histocompatibility complex class I; MHC-II, major histocompatibility complex class II; IL-10, interleukin-10; TGF-β1, transforming growth factor β1; α-MSH, alpha-melanocyte stimulating hormone; FasL, Fas ligand.

**Table 2:** Pathogenic conditions necessary for the development of AA.

	Main Features	Comments
**Loss of tolerance to self-antigens**	Self-antigens specific to the anagen phase and melanogenesis are presented by MHC-I, leading to infiltration of autoreactive CD8+ TL into the hair follicle	It has been considered the **autoimmune variant** (involving **antigen-specific pathways**) in the pathogenesis of AA. Here, CD8+ TL produce IFN-γ
**Hair follicle IP collapse inducers**	The main inducers of IP collapse are IFN-γ and SP, which are produced **nonspecifically** in response to danger signals. The latter are produced as a consequence of oxidative damage or altered hair follicle microbiome, including others (some experts consider these to be “emerging mechanisms.”)	These agents have been considered **non-immune triggers** (activate **antigen-non-specific-pathways**) in the origin of AA. In this context, IFN-γ is produced by NK, NKT, γδ-TL, and innate lymphoid cells, which express the NKG2D receptor
**Weak IP on a permanent basis**	Due to low follicular production of IP protective substances or their receptors, somewhat high levels of MHC-I or inadequate immunoregulatory properties of Treg	

AA, alopecia areata; IP, immune privilege; MHC-I, major histocompatibility complex class I; CD8+ TL, CD8+ T lymphocytes; IFN-γ, interferon-gamma; SP, substance P; NK, natural killer cell; γδ-TL, gamma-delta T lymphocytes; Treg, regulatory T-cell.

**Table 3:** Main published articles on ocular findings in alopecia areata.

Publication	Study Type and Main Findings
**Eyebrows and eyelashes**	
Insler MS et al., 1989 [90]	Case report: exclusive total madarosis of eyebrows and eyelashes in 2 sisters
Offret H et al., 1994 [91]	Case report: exclusive partial madarosis of eyelashes in 2 patients
Yoon KH et al., 1995 [92]	Case report: a 45-year-old male with alopecia universalis (AU) that started in the eyebrows but did not involve the scalp during its entire evolution
Grossman MC et al., 1996 [93]	Case report: a 30-year-old male, HIV-positive, with trichomegaly and AA of the scalp, eyebrows and, torso
Elston DM, 2002 [94]	Case report: exclusive bilateral partial madarosis of eyelashes in a 12-year-old boy
Mehta et al., 2003 [95]	Case report: exclusive bilateral partial madarosis of eyelashes in an 11-year-old girl
Grandhe NP et al., 2004 [96]	Case report: a 9-year-old girl with exclusive bilateral partial madarosis of eyelashes
Nazareth MR et al., 2009 [97] and Droubi D et al., 2012 [98]	Case report: a 3-year-old girl with bilateral trichomegaly and scalp AA. Long-term follow-up of the same case: episodic recurrences of AA along with trichomegaly
Modjtahedi BS et al., 2012 [99]	Case series: 15 patients with eyelash madarosis +/− scalp and/or eyebrow AA
De Andrade FA et al., 2014 [100]	Cross-sectional study: 54.5% of patients with AA had partial or total eyelash madarosis
Wyrwich KW et al., 2020 [101]	Interview study. 30 patients with severe or very severe AA (50% scalp hair loss): 80% experienced full or partial eyebrow and/or eyelash loss at some point during their experience of AA.
Andersen YMF et al., 2022 [102]	Cross-sectional study: 32.2% of patients had no or barely no eyelashes, 36.2% had no or barely no eyebrow hairs
Atış G et al., 2022 [103]	Case series: trichoscopic evaluation of 5 cases of exclusive eyebrow madarosis, 2 of which corresponded to AA
Doyle C et al., 2022 [104]	Case report: a 24-year-old woman with AA of linear distribution of her right frontal scalp, right medial eyebrow, and right medial eyelashes
Foad EGA et al., 2023 [105]	Prospective observational study. 60 AA patients: 35% had madarosis (*p* = 0.02). Partial loss of eyelashes separated in another group with 20% of affected patients (*p* = 0.001)
El Kissouni A et al., 2023 [106]	Case report: a 42-year-old male with exclusive total madarosis of eyelashes for the last 10 years, with spontaneous regrowth/relapse.
**Eyelids**	
Oltulu P et al., 2022 [42]	Prospective, cross-sectional study: blepharitis in 21.7% of patients with AA vs. 8% in the control group
Lin J et al., 2022 [107]	Case report: a 10-year-old boy with blepharoptosis, AA, myasthenia gravis, and goiter. Autoimmune polyglandular syndrome type II was diagnosed
Thatiparthi A et al., 2023 [108]	Retrospective cohort study: 3.91% of AA patients had inflammation of the eyelid (including blepharitis) vs. 0.85% in controls
**Lacrimal glands, conjunctiva, cornea and sclera**	
Brown AC et al., 1982 [109]	Case series of patients with AA: Krukenberg spindle (pigment deposition on the corneal endothelium) in 1 patient
Koçak Altintas AG et al., 1999 [110]	Case report: a 10-year-old boy with AA, bilateral keratoconus, and atopic keratoconjunctivitis. Hashimoto’s thyroiditis was diagnosed 3 years earlier
Chee E et al., 2015 [111]	Case series: 4 patients with AA of varying severity and dacryoadenitis
Ergin C et al., 2015 [39]	Case–control study: conjunctival papillary hypertrophy in 90% of patients. Dry eye disease (DED) in 84% of patients with AA vs. 15% in the control group
Esmer O et al., 2016 [40]	Case–control study: evaluation of dry eye occurrence in AA patients showed no significant differences with respect to the control group.
Oltulu P et al., 2022 [42]	Prospective, cross-sectional study: conjunctival papillary hypertrophy in 43.4% of AA patients vs. 12% in controls. DED was 91.3% in AA patient group: more conjunctival squamous metaplasia
Andersen YMF et al., 2022 [102]	Cross-sectional study: most patients (55.7%) did not experience irritated eyes, but 30% reported slight eye irritation. 10.3% and 4.8% had moderate and severe eye irritation, respectively
Thatiparthi A et al., 2023 [108]	Retrospective cohort study: conjunctivitis in 3.68% of patients with AA vs. 0.84% in the control group Disorders of the lacrimal system (including DED) in 7.13% of patients with AA vs. 2.63% in controls Keratitis in 1.84% of patients with AA vs. 0.62% in controls Disorders of the sclera: significantly increased risk
Foad EGA et al., 2023 [105]	Prospective observational study: 60 AA patients were included. 60% of these patients had dry eyes (*p* = 0.02)
Burgos-Blasco B et al., 2024 [112]	Case–control study: AA patients had a decreased corneal sensitivity (*p* < 0.001), and more corneal staining (*p* = 0.004). 2 eyes (4%) with a topographic diagnosis of keratoconus and another four eyes (8%) with subclinical keratoconus were detected in the AA group. No cases of keratoconus among the controls
Ma Y et al., 2025 [113]	Retrospective cohort study: prevalence of allergic conjunctivitis was higher in the AA cohort compared with controls (26% vs. 19%)
**Iris and ciliary body**	
Brown AC et al., 1982 [109]	Case series: iris color change (*n* = 3)
Haque WM et al., 2009 [114]	Case report: a 42-year-old Caucasian woman with scalp AA and idiopathic bilateral uveitis diagnosed 7 years ago. Vogt-Koyanagi-Harada (VKH) syndrome was diagnosed later.
Thatiparthi A et al., 2023 [108]	Retrospective cohort study: significantly increased risk of iridocyclitis, including uveitis
**Lens**	
Muller SA et al., 1963 [115]	Case series of patients with AU: right anterior and posterior cortical cataracts (*n* = 1), bilateral posterior subcapsular cataract (*n* = 3), bilateral unspecified cataract (*n* = 1)
Summerly R et al., 1966 [116]	Case–control study: 17% of patients with AA of varying degrees of severity had asymptomatic punctate lens opacities at cortical or posterior subcapsular level, 20% of controls with similar findings
Brown AC et al., 1982 [109]	Case series of patients with AA: right posterior lens opacity in 1 patient
Tosti A et al., 1985 [117]	Case–control study: 78% of patients with AA of varying degrees of severity had asymptomatic lens alterations: tobacco dust opacities (*n* = 36), coronary opacities (*n* = 20), light scattering (*n* = 36) vs. 27% of controls
Orecchia G et al., 1988 [118]	Case–control study: 24% of patients with AA had asymptomatic lens opacities, 25% of controls with same findings
Recupero SM et al., 1999 [12]	Case–control study: 51% of patients with AA of varying severity had asymptomatic punctate opacities (higher prevalence in AU) vs. 3% of controls
Pandhi D et al., 2009 [41]	Case–control study: 40.9% of patients with AA of varying severity and young age had lens changes: asymptomatic punctate opacities, anterior and posterior subcapsular cataracts (higher prevalence if atopic dermatitis present) vs. 11.2% of controls
De Andrade FA et al., 2014 [100]	Cross-sectional study: 18.2% of AA patients had lens changes: cataracts (*n* = 3), and pseudophakia (*n* = 1)
Ergin C et al., 2015 [39]	Case–control study: 28% of AA patients had cataracts vs. 5% of controls
Esmer O et al., 2016 [40]	Case–control study: 41.7% of eyes evaluated in young patients with AA had lens abnormalities (punctate opacities, posterior subcapsular cataract, and cortical cataract) vs. 12.2% of controls
Foad EGA et al., 2023 [105]	Prospective observational study: 60 AA patients were included. 50% of these patients had lens abnormalities (punctate opacities, posterior subcapsular cataract, and cortical cataract) (*p* = 0.001)
Burgos-Blasco B et al., 2024 [112]	Case–control study: AA patients had a more advanced cataract (*p* < 0.001): all cataracts noted were nuclear
**Vitreous humor**	
Brown AC et al., 1982 [109]	Case series of AA patients: vitreous syneresis and posterior vitreous detachment in the left vitreous cavity in 1 patient
Kalinina Ayuso V et al., 2011 [76]	Pediatric case series: coexistence of AA and idiopathic bilateral intermediate uveitis (IU) *. According to the authors’ database, they concluded a prevalence of AA of 7.5% in children with idiopathic IU.
**Choroid**	
Brown AC et al., 1982 [109]	Case series: mottled pigmentation of choroid (*n* = 1), small choroidal nevus (*n* = 1)
Pandhi D et al., 2009 [41]	Case–control study: 8.4% of AA patients had choroidal sclerosis
De Andrade FA et al., 2014 [100]	Cross-sectional study: 1 patient with 1 choroidal nevus
Thatiparthi A et al., 2023 [108]	Retrospective cohort study: there were no significant differences in the risk of chorioretinal inflammation and other disorders of the choroid in AA patients vs. controls
Şahin T et al., 2022 [119]	Case–control study: evaluation of choroidal and retinal pigment epithelium (RPE) thicknesses in 44 AA patients vs. 44 controls: there were no significant differences. However, AA patients with poor prognostic criteria had significantly thinner choroid
Oren B et al., 2023 [120]	Case–control study: choroidal thickness (CT) at the subfoveal, temporal, and nasal regions was significantly thicker in the AA group than in controls (*p* < 0.05 for all)
**Retina**	
Brown AC et al., 1982 [109]	Case series: various alterations in AA patients ranging from chorioretinal scarring (*n* = 3), vitreoretinal adhesions (*n* = 1), retinoschisis (*n* = 1), focal hypopigmentation (*n* = 2) and RPE hyperplasia (*n* = 2)
Cowan CL Jr et al., 1982 [121]	Case report: a 54-year-old black woman with progressive sensorineural hearing loss since childhood, hyperthyroidism, retinitis pigmentosa, AA, and vitiligo
Tosti A et al., 1985 [117]	Case–control study: 33% of AA patients had retinal alterations: drusen (*n* = 11) and pigmentary abnormalities (*n* = 19, lightly pigmented areas, pigmented spots, localized or diffuse hypopigmentation involving the macular area) vs. 4.5% of controls
Tosti A et al., 1986 [44]	Case–control study: mean value of the electrooculographic study significantly depressed in AA patient group, with an even greater impact on severe disease
Recupero SM et al., 1999 [12]	Case–control study: 41% of AA patients had peripheral retinal changes (pigmentary clumping, cystic/paving-stone/lattice degeneration, retinal hole, among others) vs. 23% of controls
Pandhi D et al., 2009 [41]	Case–control study: 32.5% of AA patients and young age had retinal degenerative changes (drusen, macular and lattice degeneration), pigmentary clumping and abnormal vascular changes vs. 2.5% of controls
De Andrade FA et al., 2014 [100]	Cross-sectional study. 81.4% of AA patients had: peripheral drusen, white-without-pressure changes, peripheral retinal degenerations, hyalinized vessels
Ergin C et al., 2015 [39]	Case–control study: retinopathy occurred in 18% of AA patients vs. 0% of controls
Esmer O et al., 2016 [40]	Case–control study: 33.3% of the eyes evaluated in young patients with AA had abnormalities (tigroid retina, peripapillary atrophy and macular RPE alterations) vs. 4.4% of controls
Sharma R et al., 2018 [122]	Case report: a 12-year-old boy diagnosed with AA presenting with right acute hemorrhagic retinal vasculitis
Ting HC et al., 2022 [123]	Retrospective cohort study: AA patients had significantly higher risk of developing retinal diseases, including retinal detachment, retinal vascular occlusion, and retinopathy vs. controls. In addition, the onset of retinal involvement in AA patients occurred at a younger age than those without AA
Thatiparthi A et al., 2023 [108]	Retrospective cohort study: other retinal disorders in 3.45% of AA patients vs. 2.03% in controls
Oren B et al., 2023 [120]	Case–control study: the AA and control groups did not exhibit a statistically significant difference in terms of the mean macular thicknesses, the thickness of any of the retinal layers (RPE among others), and in the peripapillary retinal nerve fiber layer (RNFL) thicknesses (*p* > 0.05 for each)
Foad EGA et al., 2023 [105]	Prospective observational study: 60 AA patients were included. 36.7% of them had statistically significant posterior segment abnormalities (tigroid retina, peripapillary atrophy, macular RPE alteration) (*p* = 0.001)
**Optic nerve**	
Lamba PA, 1969 [124]	Case report: a 13-year-old boy diagnosed with AA and finding of optic disc duplication in the left eye together with coloboma of the right iris and bilateral choroidal coloboma
Hoepf M et al., 2010 [125]	Case report: a 4-year-old boy diagnosed with left optic neuropathy who developed biopsy proven-AA shortly thereafter
De Andrade FA et al., 2014 [100]	Cross-sectional study: at the optic disc level, there were tilted discs (2 eyes), and myelinated fibers (1 eye)
Esmer O et al., 2016 [40]	Case–control study: finding of a fibrotic band extending to the optic nerve in 1 eye of 84 evaluated in 42 patients with AA vs. no such findings in the control group
**Other ocular involvement**	
Brown AC et al., 1982 [109]	Case series: bilateral exophthalmos in 1 AA patient
Fierro-Arias L et al., 2016 [126]	Cross-sectional, descriptive and observational study: no ocular alterations were found in specific ophthalmologic evaluation conducted in 29 AA patients
Nilofar F et al., 2024 [127]	Case report: a 54-year-old female patient who presented with Tolosa–Hunt Syndrome (THS), distinct patches of AA on the back of the scalp, and macular lesions on the earlobe.
	**Refractive errors**
Brown AC et al., 1982 [109]	Case series: moderate myopia in 1 AA patient
Pandhi D et al., 2009 [41]	Case–control study: in AA patients, myopia was found in 6%, and reduction in visibility to less than 3/60 was seen in 16%
Wang P et al., 2021 [128]	Case series: 6 patients with early onset high myopia and midline alopecia areata were genetically studied, finding mutations of the *COL18A1* and the *LAMA1* genes.
Foad EGA et al., 2023 [105]	Prospective observational study: 60 AA patients were included. 66.7% of them had statistically significant refractive errors (hypermetropia in 41.7% of studied eyes, astigmatism in 23.3%, and myopia in 15%) (*p* = 0.002)
Hofny ERM et al., 2024 [129]	Case–control study: errors of refraction were found in 89.2% AA patients (myopia was detected in 61.5% patients, while hypermetropia was found in 27.7%), and were significantly higher than controls (*p* = 0.027).

AA, alopecia areata; IP, immune privilege; AU, alopecia universalis; AT, alopecia totalis; TL, T lymphocytes; HIV, human immunodeficiency virus; PRO, patient-reported outcomes; DLQI, Dermatology Life Quality Index; DED, dry eye disease; VKH, Vogt-Koyanagi-Harada; IU, intermediate uveitis; CT, choroidal thickness; RPE, retinal pigment epithelium; RNFL, retinal nerve fiber layer; HLA, human leukocyte antigen; THS, Tolosa–Hunt Syndrome; SLE, systemic lupus erythematosus; IOP, intraocular pressure; OR, odds ratio; CI, confidence interval; *n*, number; aHR, adjusted hazard ratio. * The main site of inflammation is the vitreous.

**Table 4:** Key questions about ocular anterior segment involvement for dermatologists.

Ocular Pathology	Key Questions
Blepharitis	Have you experienced itchy or burning eyes? Have you ever felt heaviness or swelling in your eyelids? Do you suffer from flaking/dandruff, skin debris, or crusty eyelids? Do your eyelids stick together? Have you noticed redness on your eyelids?
Dry eye disease and keratitis	Do your eyes feel dry? Have you ever felt a gritty sensation in your eyes? Does it feel like you have a foreign body in your eyes? Do you feel pain, burning, or irritation in your eyes?/Do your eyes itch? Are your eyes sensitive to light? Do you have watery/teary eyes? Do you suffer from eye strain? Have you experienced blurred vision?/Are you experiencing vision loss? Either fluctuating vision or a sensation of cloudiness? Have you noticed redness in your eyes?
Keratoconus	Do you rub your eyes? Have you had to change your glasses frequently lately? Do you have reduced or blurred vision?
Iridocyclitis (anterior uveitis)	Do you have eye pain?/Do your eyes hurt? Have you noticed redness in your eyes? Have you experienced sensitivity or intolerance to light? Do you have decreased vision?/Have you experienced blurred vision?
Cataracts	Do you have reduced or blurred vision? (It is gradual and painless, and may be unilateral or bilateral) When you look at some kind of light, have you noticed colored halos around it? Are your eyes more sensitive to sunlight or car headlights? Have you noticed any distortion in colors perception? Have you had to change your glasses frequently lately?

**Table 5:** Key questions about ocular posterior segment involvement for dermatologists.

Ocular Pathology	Key Questions
Retinal detachment or peripheral retinal lesions	Do you see lights without any light stimulus present? Do you see floaters or black spots moving in front of your eyes? Do you see a black curtain or drape? Do you have decreased vision? (It starts at the periphery and moves toward the center. The patient may describe it as irregular, blurry, or like a curtain or veil)
Retinal vascular abnormalities	Have you ever experienced sudden, temporary loss of vision? (It is painless, and affects part or all the visual field) Have you experienced decreased vision in either eye? (This may be partial or total)
Other retinal abnormalities (e.g., retinal pigment epithelium, RPE)	Have you experienced decreased vision? Have you noticed any changes in color perception? Do you have any difficulty adjusting your vision to darkness? Do you see distorted objects or crooked straight lines?

## Data Availability

All data generated or analyzed during this study are included in this published article. Further inquiries should be directed to the corresponding authors.

## Supplementary Materials

The following supporting information can be downloaded at: https://www.mdpi.com/article/10.3390/jcm14238409/s1, Table S1: Main published articles on ocular findings in alopecia areata.

## Abbreviations

The following abbreviations are used in this manuscript:

AA	Alopecia areata
IP	Immune privilege
MHC-I	Major histocompatibility complex class I
MHC-II	Major histocompatibility complex class II
CD8+ TL	CD8+ T lymphocytes
NK	Natural killer cell
IL-10	Interleukin-10
TGF-β1	Transforming growth factor β1
α-MSH	Alpha-melanocyte stimulating hormone
IGF-1	Insulin-like growth factor 1
FasL	Fas ligand
NKG2D	Natural Killer Group 2D Receptor
KIR	Killer-cell Immunoglobulin-like Receptor
MIF	Macrophage migration inhibitory factor
MICA	MHC class I chain-related protein A
ULBP3	UL16-binding protein 3
DTH	Delayed-type hypersensitivity
Treg	Regulatory T-cell
Th2	Type 2 helper T cell
Th17	Type 17 helper T cell
VIP	Vasoactive intestinal peptide
CGRP	Calcitonin gene-related peptide
ACAID	Anterior chamber-associated immune deviation
Th1	Type 1 helper T cell
IgG1	Immunoglobulin G1
γδ Tregs	Gamma-delta regulatory T cells
iNKT	Invariant natural killer cell
IFN-γ	Interferon-gamma
SP	Substance P
HLA	Human leukocyte antigen
SI	Systemic inflammation
NO	Nitric oxide
GST	Glutathione S-transferase
AU	Alopecia universalis
AT	Alopecia totalis
TL	T lymphocytes
HIV	Human immunodeficiency virus
PRO	Patient-reported outcomes
DLQI	Dermatology Life Quality Index
DED	Dry eye disease
VKH	Vogt-Koyanagi-Harada
IU	Intermediate uveitis
CT	Choroidal thickness
RPE	Retinal pigment epithelium
RNFL	Retinal nerve fiber layer
THS	Tolosa–Hunt Syndrome
SLE	Systemic lupus erythematosus
IOP	Intraocular pressure
OR	Odds ratio
CI	Confidence interval
*n*	Number
aHR	Adjusted hazard ratio
AAPO	Alopecia Areata Patient Priority Outcomes
AASIS	Alopecia Areata Symptom Impact Scale
