# Supplementary material for: Ophthalmologic Comorbidities in Alopecia Areata
Source: J Clin Med. 2025 Nov 27;14(23):8409. doi: 10.3390/jcm14238409 (PMC12693123; doi:10.3390/jcm14238409)
Supplement: Supplementary file 1 [file jcm-14-08409-s001.zip › jcm-3983962-supplementary.pdf]

Supplementary material

Supplementary Table S1. Main published articles on ocular findings in alopecia areata.

Publication	Study type and main findings	Comments (hypotheses on pathogenesis or recommendations from authors of each article/ or our observations)
**Eyebrows and eyelashes**		
**Insler MS et al, 1989 [90]**	**Case report:** exclusive total **madarosis** of eyebrows and eyelashes in 2 sisters	Implication of genetic and emotional aspects in AA
**Offret H et al, 1994 [91]**	**Case report:** exclusive partial **madarosis** of eyelashes in 2 patients: a 9-year-old girl and a 52-year-old man	
**Yoon KH et al, 1995 [92]**	**Case report:** a 45-year-old male with alopecia universalis (AU) that started in the eyebrows but did not involve the scalp during its entire evolution	TL imbalance possibly due to infectious causes that acted as triggers of the disease
**Grossman MC et al, 1996 [93]**	**Case report:** a 30-year-old male, HIV-positive, with **trichomegaly** and **AA** of the scalp, eyebrows and, torso	Autoimmunity vs immunosuppression?
**Elston DM, 2002 [94]**	**Case report:** exclusive bilateral partial **madarosis** of eyelashes in a 12-year-old boy	Immune-mediated disorder
**Mehta et al, 2003 [95]**	**Case report:** exclusive bilateral partial **madarosis** of eyelashes in an 11-year-old girl	T-cell-mediated autoimmune reaction
**Grandhe NP et al, 2004 [96]**	**Case report:** a 9-year-old girl with exclusive bilateral partial **madarosis** of eyelashes	Autoantibodies specific to eyelash antigens
**Nazareth MR et al, 2009 [97] and Droubi D et al, 2012 [98]**	**Case report:** a 3-year-old girl with bilateral **trichomegaly** and scalp **AA**. Long-term follow-up of the same case: episodic recurrences of AA along with trichomegaly, followed by scalp hair regrowth and normal short eyelashes bilaterally	Absence of any associated medical condition, inherited disorder, or medication history
**Modjtahedi BS et al, 2012 [99]**	A **case series** of 15 patients with eyelash **madarosis** +/- scalp and/or eyebrow AA: 8 patients with isolated eyelash madarosis	Autoimmune condition
**De Andrade FA et al, 2014 [100]**	**Cross-sectional study:** 54.5% of patients with AA had partial or total eyelash **madarosis**	
**Wyrwich KW et al, 2020 [101]**	**Interview study** involving a total of 30 patients with severe or very severe AA (50% scalp hair loss): 80% of patients had experienced **full or partial eyebrow and/or eyelash loss** at some point during their experience of AA. 90% of patients considered scalp hair loss in their top three most bothersome physical signs and symptoms of AA	Other identified signs and symptoms in the top three most bothersome included **eyebrow, eyelash**, nose, body, and facial hair loss, as well as **eye irritation** and nail damage and/or appearance.
**Andersen YMF et al, 2022 [102]**	**Cross-sectional study** using data from a cohort of AA patients from the Danish Skin Cohort to provide a variety of data, including disease burden: 32.2% had **no or barely no eyelashes** and 36.2% had **no or barely no eyebrow hairs**.	It is important to point out that this study evaluated different patient-reported outcomes (PRO) such as self-perceived severity of AA symptoms, DLQI, assessment of nail appearance, eye irritation, affectation of eye lashes, involvement of eyebrows, among others

**Atış G et al, 2022 [103]**	**Case series:** trichoscopic evaluation of 5 cases of exclusive **madarosis** of the eyebrows, 2 of which corresponded to AA	Hair bulb TL infiltration
**Doyle C et al, 2022 [104]**	**Case report**: a 24-year-old woman with **AA of linear distribution** of her right frontal scalp, right medial eyebrow, and right medial eyelashes	Neuronal mechanisms, somatic mosaicism, and/or a specific migration pattern of TL
**Foad EGA et al, 2023 [105]**	**Prospective observational study:** 60 patients suffering from AA were included. 35% of these patients had **madarosis** (p = 0.02)**.** The authors separated partial loss of eyelashes in another group reporting 20% of affected patients (p = 0.001)	The authors do not specify whether madarosis involved eyebrows or eyelashes
**El Kissouni A et al, 2023 [106]**	**Case report**: a 42-year-old male with exclusive total **madarosis** of eyelashes for the last 10 years, with spontaneous regrowth/relapse. There was no history of systemic diseases, medication use, psychiatric disorders or sexually transmitted diseases.	There could be a particular group of autoantibodies directed against follicular antigens unique to eyelashes
	**Eyelids**	
**Oltulu P et al, 2022 [42]**	**Prospective, cross-sectional study: blepharitis** in 21.7% of patients with AA vs 8% in the control group	
**Lin J et al, 2022 [107]**	**Case report:** a 10-year-old boy with **blepharoptosis,** AA, myasthenia gravis, and goiter. Autoimmune polyglandular syndrome type II was diagnosed	Autoimmunity
**Thatiparthi A et al, 2023 [108]**	**Retrospective cohort study:** 3.91% of patients with AA had **inflammation of the eyelid** (including blepharitis) vs 0.85% in the control group (OR=4.75 [95% CI= 2.89-7.82])	A similar process of collapse of the IP at the ocular and hair follicle levels in AA patients
	**Lacrimal glands, conjunctiva, cornea and sclera**	
**Brown AC et al, 1982 [109]**	**Case series** of patients with AA: **Krukenberg spindle** (pigment deposition on the corneal endothelium) in 1 patient	Autoimmunity
**Koçak Altintas AG et al, 1999 [110]**	**Case report:** a 10-year-old boy with AA, **bilateral keratoconus**, and atopic keratoconjunctivitis. Hashimoto's thyroiditis was diagnosed 3 years earlier	The association between atopy and AA, and atopy with keratoconus should be considered, especially in patients with visual symptoms
**Chee E et al, 2015 [111]**	**Case series:** 4 patients with AA of varying severity and **dacryoadenitis**	Genetic susceptibility and attenuation of IP in lacrimal gland and hair follicle
**Ergin C et al, 2015 [39]**	**Case-control study: conjunctival papillary hypertrophy** in 90% of patients.<br>**Dry eye disease (DED)** in 84% of patients with AA vs 15% in the control group: higher scores on corneal staining stage (CSS) test indicating ocular surface abnormalities	It is usually a feature of allergic conjunctivitis<br>Multifactorial: autoimmunity may play a major role
**Esmer O et al, 2016 [40]**	**Case-control study:** evaluation of dry eye occurrence in patients with AA in the study showed no significant differences with respect to the control group.	The authors only used the Schirmer test to assess ocular surface and dry eye symptoms
**Oltulu P et al, 2022 [42]**	**Prospective, cross-sectional study: conjunctival papillary hypertrophy** in 43.4% of patients with AA vs 12% in the control group	Inflammation and apoptosis

	The prevalence of **DED** was 91.3% in AA patient group: more **conjunctival squamous metaplasia** than normal	Alteration in tear stability due to loss of goblet cells. Similar pathogenesis in AA and DED
**Andersen YMF et al, 2022 [102]**	**Cross-sectional study** using data from a cohort of AA patients from the Danish Skin Cohort to provide a variety of data, including disease burden: Overall, most patients (55.7%) did not experience irritated eyes, but 30% reported slight **eye irritation**. 10.3% and 4.8% had moderate and severe eye irritation, respectively	This study evaluated different patient-reported outcomes (PRO) such as self-perceived severity of AA symptoms, DLQI, assessment of nail appearance, eye irritation, among others
**Thatiparthi A et al, 2023 [108]**	**Retrospective cohort study: conjunctivitis** in 3.68% of patients with AA vs 0.84% in the control group (OR=4.52 [95% CI= 2.71-7.55]). **Disorders of the lacrimal system** (including DED) in 7.13% of patients with AA vs 2.63% in the control group (OR=2.84 [95% CI= 1.96-4.11]).<br>**Keratitis** in 1.84% of patients with AA vs 0.62% in the control group (OR=3.01 [95% CI= 1.47-6.12]). Significantly increased risk of having **disorders of the sclera** (OR=6.28 [95% CI= 1.48-26.52])	A similar process of collapse of the IP at the ocular and hair follicle levels in AA patients
**Foad EGA et al, 2023 [105]**	**Prospective observational study:** 60 patients suffering from AA with varying degrees of severity were included. 60% of these patients had **dry** eyes (p = 0.02)	
**Burgos-Blasco B et al, 2024 [112]**	**Case-control study:** AA patients had a **decreased corneal sensitivity** (*p* < 0.001), and **more corneal staining** (*p* = 0.004).<br>**Corneal topographic and biomechanical parameters** were altered in AA patients.<br>Two eyes (4%) with a topographic diagnosis of **keratoconus** and another four eyes (8%) with subclinical keratoconus were detected in the AA group. No cases of clinical or subclinical keratoconus were noted among the controls	-Causes of reduced corneal sensitivity: 1.DED, specifically in the aqueous tear deficiency subset. 2. Chronic inflammation induced by tear dysfunction<br>-There could be a higher risk of keratoconus in AA patients: a key role of the immune system in the pathogenesis of keratoconus is postulated, which could be relevant to its development in AA<br>- Routine ophthalmological examination is suggested (see below)
**Ma Y et al, 2025 [113]**	A **retrospective cohort study** that evaluated the prevalence and incidence rates of comorbidities in patients with AA, vitiligo, atopic dermatitis and psoriasis. In the AA cohort the prevalence of **allergic conjunctivitis** was higher compared with the matched controls (26% vs 19%)	It is important to raise awareness of the risk of comorbidities in patients with skin diseases, which in turn can guide medical decisions
	**Iris and ciliary body**	
**Brown AC et al, 1982 [109]**	**Case series** of patients with AA**: iris color change** (n=3)	Autoimmunity
**Haque WM et al, 2009 [114]**	**Case report:** a 42-year-old Caucasian woman with scalp AA and **idiopathic bilateral uveitis** diagnosed 7 years ago. Before uveitis, the patient experienced a febrile illness with meningismus, headaches, and dysacusia. With this set of findings, the authors diagnosed Vogt-Koyanagi-Harada (VKH) syndrome	The authors propose that hair loss in VKH syndrome is due to AA, and this association would be consistent with a common pathophysiology: genetic susceptibility and autoimmunity

**Thatiparthi A et al, 2023 [108]**	**Retrospective cohort study: iridocyclitis,** including uveitis (OR=4.30 [95% CI= 1.34-13.78])	Collapse of ocular IP
	**Lens**	
**Muller SA et al, 1963 [115]**	**Case series** of patients with AU**:** right anterior and posterior **cortical cataracts** (n=1), bilateral **posterior subcapsular cataract** (n=3), bilateral unspecified cataract (n=1)	Association between severe or persistent AA with lenticular changes. Recommendation: routine slit lamp examination of the lenses in AU and in repeated episodes of extensive AA
**Summerly R et al, 1966 [116]**	**Case-control study:** 17% of patients with AA of varying degrees of severity had **punctate lens opacities** at cortical or posterior subcapsular level, 20% of controls with similar findings	High incidence of similar minor lens changes in the healthy population. Routine slit lamp examination is not recommended in AA, unless vision impairment is reported
**Brown AC et al, 1982 [109]**	**Case series** of patients with AA**:** right **posterior lens opacity** in 1 patient	No specific comment in the study
**Tosti A et al, 1985 [117]**	**Case-control study:** 78% of patients with AA of varying degrees of severity had asymptomatic lens alterations: tobacco dust **opacities** (n=36), coronary **opacities** (n=20), light scattering (n=36) vs 27% of the control group	The clinical significance of this finding cannot be determined, follow-up is required
**Orecchia G et al, 1988 [118]**	**Case-control study:** 24% percent of patients with AA had asymptomatic lens **opacities**, 25% of controls with the same findings, and there was no significant correlation	There is no significant clinical relevance to these findings in AA
**Recupero SM et al, 1999 [12]**	**Case-control study:** 51% of patients with AA of varying severity had asymptomatic **punctate opacities** (higher prevalence in AU) vs 3% of the control group	Skin and lens have a common embryologic origin (ectoderm), which could explain their pathogenic association
**Pandhi D et al, 2009 [41]**	**Case-control study:** 40.9% of patients with AA of varying severity and young age had lens changes: asymptomatic **punctate opacities**, **anterior and posterior subcapsular cataracts** (higher prevalence if atopic dermatitis present) vs 11.2% of the control group	Autoimmunity and oxidative stress as causative factors in AA with lenticular involvement. Every AA patient, irrespective of age and extent of disease, should undergo complete and routine ophthalmological examination
**De Andrade FA et al, 2014 [100]**	**Cross-sectional study:** 18.2% of patients with AA of varying degrees of severity had lens changes: **cataracts** (n=3), and **pseudophakia** (n=1)	Patients with associated autoimmune diseases more frequently had AU and multiple ocular abnormalities
**Ergin C et al, 2015 [39]**	**Case-control study:** 28% of patients with AA of varying degrees of severity had **cataracts** vs 5% of the control group	Refer all patients with AA to an ophthalmologist for evaluation of ocular pathologies
**Esmer O et al, 2016 [40]**	**Case-control study:** 41.7% of eyes evaluated in young patients with AA of varying degrees of severity had lens abnormalities **(punctate opacities, posterior subcapsular cataract, and cortical cataract)** vs 12.2% of the control group	There were no patients with associated atopic dermatitis in the study, which excludes it as a causal factor of lenticular involvement in this study
**Foad EGA et al, 2023 [105]**	**Prospective observational study:** 60 patients suffering from AA with varying degrees of severity were included. 50% of these patients had lens abnormalities **(punctate opacities, posterior subcapsular cataract, and cortical cataract)** ($p = 0.001$)	

**Burgos-Blasco B et al, 2024 [[112]]**	**Case-control study:** AA patients had a more **advanced cataract** (*p* < 0.001): all cataracts noted were **nuclear**	Routine ophthalmological examination is suggested if one or more of the following are present: ocular symptoms, other diseases (e.g., atopic dermatitis), severe AA (AU, AT)
	**Vitreous humor**	
**Brown AC et al, 1982 [[109]]**	**Case series** of patients with AA**: vitreous syneresis and posterior vitreous detachment** in the left vitreous cavity in 1 patient	Autoimmunity
**Kalinina Ayuso V et al, 2011 [[76]]**	Pediatric **case series:** coexistence of AA and **idiopathic bilateral intermediate uveitis** (IU)*. According to the authors' database, they concluded a prevalence of AA of 7.5% in children with idiopathic IU.<br>*The main site of inflammation is the vitreous.	An autoimmune basis in both conditions (IP collapse) in addition to genetic and environmental factors (substance P)
	**Choroid**	
**Brown AC et al, 1982 [[109]]**	**Case series** of patients with AA**: mottled pigmentation** of choroid (n=1), small choroidal nevus (n=1)	Autoimmunity
**Pandhi D et al, 2009 [[41]]**	**Case-control study:** 8.4% of AA patients had **choroidal sclerosis**	
**De Andrade FA et al, 2014 [[100]]**	**Cross-sectional study:** 1 patient with 1 **choroidal nevus**	
**Thatiparthi A et al, 2023 [[108]]**	**Retrospective cohort study:** there were no significant differences in the risk of chorioretinal inflammation and other disorders of the choroid in AA patients vs controls	
**Şahin T et al, 2022 [[119]]**	**Case-control study:** evaluation of choroidal and retinal pigment epithelium (RPE) thicknesses in 44 patients with AA vs 44 controls: there were no significant differences. However, AA patients with poor prognostic criteria had **significantly thinner choroid**	Choroidal thickness monitoring may provide information on disease prognosis in patients with AA
**Oren B et al, 2023 [[120]]**	**Case-control study: choroidal thickness** (CT) at the subfoveal, temporal, and nasal regions **was significantly thicker** in the AA group than in the control group (p<0.05 for all)	The increase in CT in AA patients may be due to inflammation caused by TL activity toward components of hair follicles as well as choroidal melanocytes. However, the patients in the study were newly diagnosed, so it is possible that CT may change in the long-term follow-up of AA patients
	**Retina**	
**Brown AC et al, 1982 [[109]]**	**Case series** of patients with AA**:** various alterations in patients with AA ranging from **chorioretinal scarring** (n=3), **vitreoretinal adhesions** (n=1), **retinoschisis** (n=1), **focal hypopigmentation** (n=2) and **RPE hyperplasia** (n=2)	Autoimmunity. Finding of associated testicular anomalies: oligo- or aspermia, hypogonadism, epididymal cyst and hyper retractile testis

**Cowan CL Jr et al, 1982 [[121]]**	**Case report:** a 54-year-old black woman with progressive sensorineural hearing loss since childhood, hyperthyroidism, **retinitis pigmentosa**, AA, and vitiligo	Immunological disturbances have been implicated in these disorders
**Tosti A et al, 1985 [[117]]**	**Case-control study:** 33% of patients with AA had retinal alterations: **drusen** (n=11) and **pigmentary abnormalities** (n=19, lightly pigmented areas, pigmented spots, localized or diffuse hypopigmentation involving the macular area) vs 4.5% of the control group	Cell damage in AA may involve melanocytes of the hair follicle and pigment cells of RPE
**Tosti A et al, 1986 [[44]]**	**Case-control study: mean value of the electrooculographic study significantly depressed** in AA patient group, with an even greater impact in severe disease	A dysfunction of the RPE is postulated that could be due to pigment cell damage by a primary or secondary insult
**Recupero SM et al, 1999 [[12]]**	**Case-control study:** 41% of patients with AA had **peripheral retinal changes** (pigmentary clumping, cystic/paving-stone/lattice degeneration, retinal hole, among others) vs 23% of the control group	Autoimmunity that could be directed against hair melanocytes and pigment cells of RPE. Ophthalmologic evaluation is recommended
**Pandhi D et al, 2009 [[41]]**	**Case-control study:** 32.5% of patients with AA and young age had **retinal degenerative changes** (drusen, macular and lattice degeneration), **pigmentary clumping** and **abnormal vascular changes** vs 2.5% of the control group	Pigment cell dysfunction in the pathogenesis of AA and retinal involvement. Routine ophthalmologic evaluation
**De Andrade FA et al, 2014 [[100]]**	**Cross-sectional study**. 81.4% of patients with AA of varying degrees of severity presented: **peripheral drusen, white-without-pressure changes, peripheral retinal degenerations, hyalinized vessels**	Patients with associated autoimmune diseases more frequently had AU and multiple ocular abnormalities
**Ergin C et al, 2015 [[39]]**	**Case-control study: retinopathy** occurred in 18% of patients with AA of varying degrees of severity vs 0% of the control group	Ophthalmologic assessment of all patients with AA for evaluation of ocular pathologies
**Esmer O et al, 2016 [[40]]**	**Case-control study:** 33.3% of the eyes evaluated in young patients with AA of varying severity had abnormalities (**tigroid retina, peripapillary atrophy and macular RPE alterations**) vs 4.4% of the control group	Autoimmunity may play an important role in both AA and RPE abnormalities
**Sharma R et al, 2018 [[122]]**	**Case report:** a 12-year-old boy diagnosed with AA presenting with right **acute hemorrhagic retinal vasculitis**	The only workup finding was a positive HLA-B27
**Ting HC et al, 2022 [[123]]**	**Retrospective cohort study:** patients with AA had significantly higher risk of developing **retinal diseases** (aHR 3.10 [95% CI= 2.26-4.26]), including **retinal detachment**, **retinal vascular occlusion, and retinopathy** vs controls. In addition, the onset of retinal involvement in patients with AA occurred at a younger age than those without AA	Pathogenesis of AA and retinal diseases may be due to pigment cell injury (hair follicle melanocytes and RPE cells) and systemic inflammation
**Thatiparthi A et al, 2023 [[108]]**	**Retrospective cohort study: other retinal disorders** in 3.45% of patients with AA vs 2.03% in the control group (OR=1.72 [95% CI= 1.02-2.89])	Collapse of ocular IP similar to that of hair

**Oren B et al, 2023 [[120]]**	**Case-control study:** the AA and control groups did not exhibit a statistically significant difference in terms of the mean macular thicknesses, the thickness of any of the retinal layers (RPE among others), and in the peripapillary retinal nerve fiber layer (RNFL) thicknesses (p>0.05 for each)	Patients in the study were newly diagnosed, so it is possible to see a change in RPE thickness with an increase in inflammation. Absence of pigment cells in the macula and RNFL could explain the absence of changes in the thickness of these tissues
**Foad EGA et al, 2023 [[105]]**	**Prospective observational study:** 60 patients suffering from AA with varying degrees of severity were included. 36.7% of these patients had statistically significant posterior segment abnormalities **(tigroid retina, peripapillary atrophy, and macular RPE alteration)** (p = 0.001)	
**Optic nerve**		
**Lamba PA, 1969 [[124]]**	**Case report:** a 13-year-old boy diagnosed with AA and finding of **optic disc duplication** in the left eye together with coloboma of the right iris and bilateral choroidal coloboma	
**Hoepf M et al, 2010 [[125]]**	**Case report:** a 4-year-old boy diagnosed with left **optic neuropathy** who developed biopsy proven-AA shortly thereafter	Inflammation is proposed as a causal factor in both entities
**De Andrade FA et al, 2014 [[100]]**	**Cross-sectional study:** at the optical disk level, there were **tilted discs** (2 eyes) y **myelinated fibers** (1 eye)	
**Esmer O et al, 2016 [[40]]**	**Case-control study:** finding of a **fibrotic band extending to the optic nerve** in 1 eye of 84 evaluated in 42 patients with AA vs. no such findings in the control group	Autoimmunity may play an important role in both AA and ocular abnormalities
**Other ocular involvement**		
**Brown AC et al, 1982 [[109]]**	**Case series: bilateral exophthalmos** in 1 patient with AA	Thyroid disease, muscular alterations, and orbital masses were ruled out
**Fierro-Arias L et al, 2016 [[126]]**	**Cross-sectional, descriptive and observational study:** no ocular alterations were found in specific ophthalmologic evaluation conducted in 29 patients with AA.	New studies are required with a larger number of patients, and longer follow-up periods.
**Nilofar F et al, 2024 [[127]]**	**Case report:** a 54-year-old female patient who presented with **Tolosa-Hunt Syndrome** (THS), distinct patches of AA on the back of the scalp, and macular lesions on the earlobe. Moreover, further studies confirmed the presence of systemic lupus erythematosus (SLE)	THS is a rare inflammatory condition characterized by **painful ophthalmoplegia**.<br>Autoimmunity is postulated as the basis for the clinical manifestations of this case
	**Refractive errors**	
**Brown AC et al, 1982 [[109]]**	**Case series: moderate myopia** in 1 AA patient	
**Pandhi D et al, 2009 [[41]]**	**Case-control study:** in AA patients**, myopia** was found in 6%, and **reduction of visibility** to less than 3/60 was seen in 16%	
**Wang P et al, 2021 [[128]]**	**Case series:** 6 patients with early onset **high myopia** and midline alopecia areata were genetically studied, finding mutations of the *COL18A1* and the *LAMA1* genes. Further	All 6 patients had aplasia cutis: in the study there was no clear distinction

	assessments indicated that patients with *COL18A1* mutations had Knobloch syndrome, and the patients with *LAMA1* mutations had Poretti–Boltshauser syndrome	between lesions of the latter entity and AA lesions
**Foad EGA et al, 2023 [105]**	**Prospective observational study:** 60 patients suffering from AA with varying degrees of severity were included. 66.7% of these patients had statistically significant **refractive errors** (hypermetropia in 41.7 % of studied eyes, astigmatism in 23.3 %, and myopia in 15%) (p = 0.002)	The authors recommend that patients with alopecia undergo routine ophthalmologic examinations to evaluate their refraction
**Hofny ERM et al, 2024 [129]**	**Case-control study: errors of refraction** were found in 89.2% AA patients (myopia in one or both eyes was detected in 61.5% patients, while hypermetropia in one or both eyes was found in 27.7% patients), and were significantly higher than controls (p = 0.027). Nevertheless, no statistically significant differences were found between intraocular pressure (IOP), fundus, anterior segment changes, and madarosis in patients vs controls	The relationship between AA and ocular changes could be explained by common etiologic factors, such as oxidative stress and autoimmune destruction of hair follicles and various ocular structures. The authors recommend a complete ophthalmologic examination as part of the treatment algorithm for all patients with AA.

AA, alopecia areata; IP, immune privilege; AU, alopecia universalis; AT, alopecia totalis; TL, T lymphocytes; HIV, human immunodeficiency virus; PRO, patient-reported outcomes; DLQI, Dermatology Life Quality Index; DED, dry eye disease; VKH, Vogt-Koyanagi-Harada; IU, intermediate uveitis; CT, choroidal thickness; RPE, retinal pigment epithelium; RNFL, retinal nerve fiber layer; HLA, human leukocyte antigen; THS, Tolosa-Hunt Syndrome; SLE, systemic lupus erythematosus; IOP, intraocular pressure; OR, odds ratio; CI, confidence interval; n, number; aHR, adjusted hazard ratio
